# Developing a Standardized and Reusable Method to Link Distributed Health Plan Databases to the National Death Index: Methods Development Study Protocol

**DOI:** 10.2196/21811

**Published:** 2020-11-02

**Authors:** Candace C Fuller, Wei Hua, Charles E Leonard, Andrew Mosholder, Ryan Carnahan, Sarah Dutcher, Katelyn King, Andrew B Petrone, Robert Rosofsky, Laura A Shockro, Jessica Young, Jea Young Min, Ingrid Binswanger, Denise Boudreau, Marie R Griffin, Margaret A Adgent, Jennifer Kuntz, Cheryl McMahill-Walraven, Pamala A Pawloski, Robert Ball, Sengwee Toh

**Affiliations:** 1 Department of Population Medicine Harvard Pilgrim Health Care Institute Harvard Medical School Boston, MA United States; 2 Office of Surveillance and Epidemiology Center for Drug Evaluation and Research Food and Drug Administration Silver Spring, MD United States; 3 Center for Pharmacoepidemiology Research and Training Department of Biostatistics, Epidemiology, and Informatics Perelman School of Medicine, University of Pennsylvania Philadelphia, PA United States; 4 University of Iowa, College of Public Health Iowa City, IA United States; 5 Health Information Systems Consulting Milton, MA United States; 6 Vanderbilt University Nashville, TN United States; 7 Kaiser Permanente Colorado Aurora, CO United States; 8 Kaiser Permanente Washington Health Research Institute and University of Washington Seattle, WA United States; 9 Kaiser Permanente Northwest Portland, OR United States; 10 Aetna, a CVS Health company Blue Bell, PA United States; 11 HealthPartners Institute Bloomington, MN United States

**Keywords:** National Death Index, data linkage, all-cause mortality, cause specific mortality, distributed analysis, multisite research

## Abstract

**Background:**

Certain medications may increase the risk of death or death from specific causes (eg, sudden cardiac death), but these risks may not be identified in premarket randomized trials. Having the capacity to examine death in postmarket safety surveillance activities is important to the US Food and Drug Administration’s (FDA) mission to protect public health. Distributed networks of electronic health plan databases used by the FDA to conduct multicenter research or medical product safety surveillance studies often do not systematically include death or cause-of-death information.

**Objective:**

This study aims to develop reusable, generalizable methods for linking multiple health plan databases with the Centers for Disease Control and Prevention’s National Death Index Plus (NDI+) data.

**Methods:**

We will develop efficient administrative workflows to facilitate multicenter institutional review board (IRB) review and approval within a distributed network of 6 health plans. The study will create a distributed NDI+ linkage process that avoids sharing of identifiable patient information between health plans or with a central coordinating center. We will develop standardized criteria for selecting and retaining NDI+ matches and methods for harmonizing linked information across multiple health plans. We will test our processes within a use case comprising users and nonusers of antiarrhythmic medications.

**Results:**

We will use the linked health plan and NDI+ data sets to estimate the incidences and incidence rates of mortality and specific causes of death within the study use case and compare the results with reported estimates. These comparisons provide an opportunity to assess the performance of the developed NDI+ linkage approach and lessons for future studies requiring NDI+ linkage in distributed database settings. This study is approved by the IRB at Harvard Pilgrim Health Care in Boston, MA. Results will be presented to the FDA at academic conferences and published in peer-reviewed journals.

**Conclusions:**

This study will develop and test a reusable distributed NDI+ linkage approach with the goal of providing tested NDI+ linkage methods for use in future studies within distributed data networks. Having standardized and reusable methods for systematically obtaining death and cause-of-death information from NDI+ would enhance the FDA’s ability to assess mortality-related safety questions in the postmarket, real-world setting.

**International Registered Report Identifier (IRRID):**

DERR1-10.2196/21811

## Introduction

### Public Health Significance and Study Motivation

Certain medications may increase the risk of death and specific causes of death (eg, sudden cardiac death [SCD]), but these risks may not be identified in premarket randomized controlled trials owing to the relatively small sample sizes and the highly selected patient populations in these trials. The capacity to examine the risk of death in postmarket safety surveillance activities is an important part of the US Food and Drug Administration’s (FDA) mission to protect public health. Although the FDA Adverse Event Reporting System (FAERS) [1] identifies drug safety signals [2] and is vital to this mission [3], FAERS has a number of known limitations. Similar to most spontaneous reporting systems that rely primarily on voluntarily reported adverse events, FAERS is susceptible to underreporting, variable data quality, lack of denominator information, and frequent absence of details necessary to evaluate clinical events and associations with a specific medication [4-6].

Other components of the FDA’s postmarket medical product safety surveillance system complement FAERS in many ways but often do not systematically capture death or cause-of-death information. For example, the FDA’s Sentinel System [7,8] includes a distributed network of electronic health plan databases. The health plans that participate in the Sentinel System or other multicenter research networks routinely capture data on in-hospital deaths and medically attended deaths but often do not have complete capture of out-of-hospital deaths or cause-of-death information. Although some health plans perform routine or ad hoc linkages with local or state death registries or Social Security Administration (SSA) data to address these data gaps, such linkages are often specific to a particular study or site.

In addition, some multicenter research networks use a distributed data approach in which individual study sites or health plans maintain physical and operational control over their electronic health data behind their respective firewalls. A distributed network approach promotes data sharing by protecting patient privacy, data security, and proprietary interests [9-12]. The development of a systematic method to link distributed databases to a data source that includes both death and cause-of-death information, such as the National Death Index (NDI), would enhance the FDA’s ability to answer mortality-related safety questions in the postmarket setting.

### NDI and Cause-of-Death Information

The NDI, a self-supporting service within the National Center for Health Statistics (NCHS) of the Centers for Disease Control and Prevention, is a centralized database of death record information compiled from the vital statistics offices of states and other jurisdictions. The NDI provides death information including death date and death certificate number (referred to as the NDI data) and cause of death from death certificates (referred to as NDI Plus or NDI+ data) upon request [13]. Although the SSA also provides the fact of death, it does not provide cause-of-death information, and a 2011 determination by the SSA that data submitted electronically by states cannot be publicly shared in the SSA death master file has since limited its coverage [14].

The limitations of the cause-of-death information derived from death certificates, the foundation of state death records, and subsequent NDI information have been well described [15]. In brief, although efforts have been made to improve the completeness and accuracy of cause-of-death reporting in the United States, the cause-of-death information in the death certificate ultimately represents medical opinions. The certifier (eg, attending physician, medical examiner, coroner) provides a clinical judgment informed by their training, knowledge of medicine, and available medical history of the decedent [16]. Certifier requirements (eg, coroner or medical examiner) can also vary according to state laws [17]. Variation in all of these elements can lead to inaccurate documentation by the certifier, and studies have found that causes of death listed on the death certificates, and subsequently coded in NDI+ data, may be misclassified by 16% to 40%, depending on the cause [18,19]. Misclassification may increase when the death is sudden and unobserved [20,21] and also when more narrowly defined causes of death are listed [22]. Errors introduced during translation of the causes of death on death certificates to the International Classification of Diseases, 10th Revision (ICD-10) codes are much less common [23,24].

Despite the known limitations of death certificate data, researchers have used these data to examine national death data trends and changes in causes of death over time [22,25,26] and have used death certificate data with other data sources to more accurately define specific causes of death, such as SCD [27]. Notwithstanding the above mentioned limitations, the NDI is currently the only complete national source of death and cause-of-death information accessible to large-scale population-based epidemiologic studies in the United States.

### Primary and Secondary Objective of the Study

#### Overview of the Study Objectives

The primary objective of this study is to develop reusable administrative and technical processes for linking multiple health plan databases with NDI+ data to allow the FDA to assess death and specific causes of death as outcomes in medical product safety and effectiveness studies in distributed networks of electronic health plan databases. We will pilot the developed approach through a use case comprising antiarrhythmic medication users and nonusers. The outcomes of interest in the use case are all-cause mortality and SCD, but cardiovascular death may also be examined if it is feasible within the study timeline.

The secondary objectives focus on using the linked health plan and NDI+ data to estimate the incidences and incidence rates of mortality and specific causes of death within the use case and comparing them with estimates reported in the literature. Examining the incidences and incidence rates of mortality and death from specific causes within the use case will provide an opportunity to assess the performance of the workflows and processes developed under the primary objectives.

#### Primary Objectives

1. Develop and pilot an administrative workflow that facilitates efficient, coordinated, multicenter institutional review board (IRB) review and approval for linking health plan data with NDI+ data.
2. Create and pilot a distributed technical process for linking health plan and NDI+ data that:
  uniformly identifies records to be submitted to the NDI from each health plan
  avoids sharing of identifiable patient information between participating health plans or with the coordinating center and allows health plans to work directly with the NDI
  uses standardized criteria to select and retain confirmed or best match from linked NDI+ data across multiple health plans
  harmonizes linked information across multiple health plans by saving NDI+ data in a standardized format

#### Secondary Objectives

The secondary objectives are as follows:

1. Estimate the incidences and incidence rates of all-cause mortality, SCD, and potentially cardiovascular death within a high-risk use case cohort (ie, individuals using antiarrhythmic medications) and an average-risk cohort (ie, individuals not on antiarrhythmic medications).
2. Assess the performance of the developed workflows and processes for linking health plan and NDI+ data by examining the incidences and incidence rates of all-cause mortality, SCD, and potentially cardiovascular death within the use case cohorts, and comparing them with estimates previously reported in the literature.

**Figure 1:**
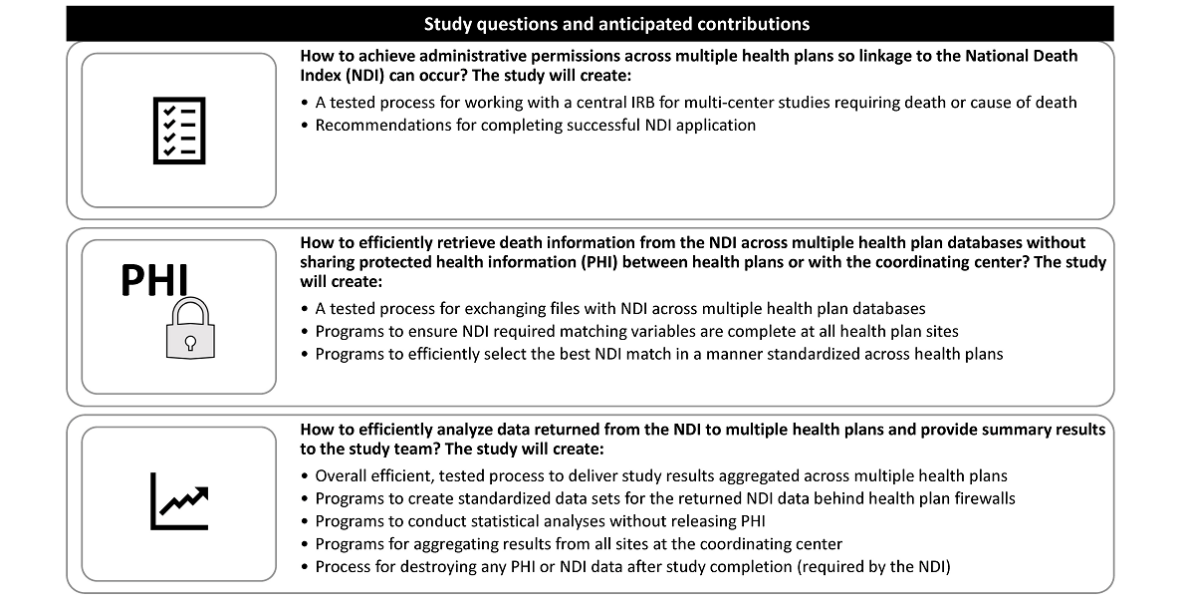
Overview of study questions and anticipated contributions. NDI: National Death Index; IRB: Institutional Review Board; PHI: Protected Health Information.

Figure 1 provides an overview of the questions this study will address and anticipated contributions.

## Methods

### Use Case and Rationale

For this study, we chose antiarrhythmic medications as the use case. The arrhythmogenicity of antiarrhythmic medications is well known, and several antiarrhythmic medications are known to be associated with elevated risks of all-cause mortality and SCD [28-30]. SCD associated with arrest, generally defined as the sudden cessation of heart function, is a major cause of mortality and a major public health concern. Ventricular fibrillation is often associated with SCD and is a pulseless arrhythmia with irregular and chaotic electrical activity and ventricular contraction in which the heart immediately loses its ability to pump [31]. Ventricular fibrillation is the initial electrocardiogram rhythm in 75% of outpatient cases of SCD [32]. Torsade de Pointes is a specific form of polymorphic ventricular tachycardia that if rapid or prolonged can lead to ventricular fibrillation and SCD [33].

There are approximately 20 cardiovascular medications and well over 100 noncardiovascular medications suspected of causing SCD, ventricular fibrillation, or Torsade de Pointes [28]. For example, although class III antiarrhythmic medications are used to treat atrial or ventricular arrhythmias, they prolong repolarization and cardiac refractoriness and can increase an individual’s propensity for Torsade de Pointes [34]. In addition, individuals with arrhythmias are at a high risk of death and SCD. Therefore, we expect all-cause mortality as well as SCD to be more common in antiarrhythmic medication users than in a cohort not exposed to these medications. As the incidences of mortality and SCD in the US population are well described [35-37], identification of a cohort at average risk of these outcomes will provide an efficient reference point for antiarrhythmic medication users and an opportunity to assess the performance or *validity* of the linkage to NDI+ data.

### Participating Organizations

This project will be led and coordinated by the Harvard Pilgrim Health Care Institute (HPHCI), which will work closely with the FDA and participating health plans in all aspects of the project. A total of 6 health plans—Aetna, a CVS Health company; HealthPartners Institute; Kaiser Permanente Colorado; Kaiser Permanente Northwest; Kaiser Permanente Washington; and Vanderbilt University (which provides access to Tennessee Medicaid data)—will participate in this project. They represent a diverse group of health plans, including national insurers, regional health plans, and integrated delivery systems, and cover both commercial and public insurance programs. Although the project will leverage the Sentinel infrastructure and be built on the successful collaboration among participating institutions, it will be conducted outside of the Sentinel Initiative and will be relevant to other distributed data networks. The project is a research activity subject to the Office for Human Research Protections regulations, following the 45 Code of Federal Regulations 46 [38] on the protection of human subjects, and will undergo IRB review.

### Development of Multisite Administrative Workflows to Support Linkage to NDI+ Data

#### Overview of the Administrative Workflows

This project will develop reusable and flexible administrative workflows required to support simultaneous linkage of multiple health plan databases with NDI+ data. As the lead project site and coordinating center, the HPHCI will develop and facilitate administrative processes for IRB workflow as well as submission of the master NDI application on behalf of the participating health plans. The HPHCI will lead the development of the NDI application package, coordinate review by participating health plans and the FDA as well as the execution of legal agreements (as necessary), and will submit the master NDI application that will include IRB documents and approvals.

The HPHCI will review, consider, and accommodate the requirements of institutions involved in this project to ensure that the developed workflows for NDI and IRB application review and approval are flexible enough to be reused in future studies. This may require review and response to any of the following: health plan institutional requirements, FDA requirements, relevant federal requirements (eg, revised Common Rule [39] and other requirements), relevant state or local jurisdiction requirements (eg, laws concerning death data), institutional IRB requirements, or NCHS/NDI requirements. For example, preliminary work with participating health plans suggested the need to consider any state or local laws pertaining to death data within project workflows. Balancing such requirements as well as any other identified prerequisites or constraints will be a key focus of the developed multisite administrative workflow. In the following paragraphs, we describe our anticipated processes for implementing coordinated multisite, central IRB review and approval, as well as multisite NDI application review and approval.

#### IRB Application Workflow

The revised Common Rule requires the use of a central IRB for multisite research, with certain exceptions (82 Fed. Reg. at 7265 [final rule §.114]) [39]. In addition, the NDI currently requires all studies requesting NDI+ data to undergo IRB review. This project will develop and pilot an administrative workflow that facilitates efficient, coordinated, multicenter IRB review and approval for linking health plan data with NDI+ data in accordance with the revised Common Rule.

The IRB at Harvard Pilgrim Health Care, the parent organization of the HPHCI, is responsible for managing and supporting scientific and ethical review of research studies submitted by the HPHCI. The HPHC IRB also enters into reliance agreements for multisite studies as a reviewing IRB and a relying IRB. The HPHC IRB holds a Federalwide Assurance (FWA) with the US Department of Health and Human Services [FWA00000100] and thus is compliant with human subjects regulations within 45 Code of Federal Regulations 46 [38,40]. As the lead study institution, the HPHCI will aim to have the HPHC IRB serve as the IRB of record, with all participating sites ceding their IRB review to the HPHC IRB. However, if the use of a single IRB entity is determined not to be feasible or acceptable to the NCHS, the NDI board, or participating health plans, the HPHCI will work with each participating health plan to attain IRB approval.

The study team will describe the necessary administrative workflow processes and highlight any encountered governance challenges (eg, local institutional policies or procedures) and potential solutions. Furthermore, the study team will address any complications with individual study sites obtaining approval to cede to the HPHC IRB in the final developed workflow. The anticipated central IRB workflow is as follows:

1. The HPHCI will submit an IRB application to the HPHC IRB and obtain HPHC IRB approval for the study. The HPHCI and collaborating health plans to cede review by initiating and executing reliance agreements with respective health plan IRB(s). Reliance agreements must be in place for local health plan IRBs to cede review and for the HPHC IRB to serve as the lead reviewing IRB. We anticipate the cede process will proceed as follows:
  The HPHCI will provide the HPHC IRB application and approval to participating health plans for review. The HPHCI will work with health plans to address any concerns or amendments needed to satisfy approval to cede to the HPHC IRB. Individual health plan–specific policies and procedures may apply and will be documented.
  Participating health plans will prepare all necessary cede request documents required by site IRB(s) and the HPHC IRB. Health plans will submit a cede request to the HPHC IRB.
  The HPHC IRB will review the submitted cede requests and may require additional health plan–specific materials in determining approval to accept the request (eg, documentation of human subjects training from key personnel).
  The lead HPHC IRB and the IRB(s) at participating health plans will fully execute reliance agreements, formally known as IRB authorization agreements, to officially confirm the HPHC IRB as the lead reviewing IRB of record for the study.
2. Following the completed cede process, the HPHC IRB will be responsible for continuing review as well as amendment and reviewing of any unanticipated problems. Participating health plans will be responsible for timely communication and reporting to the HPHC IRB for any unanticipated problems encountered at their site for this study.

The anticipated central IRB workflow process will be updated as new procedures or processes are encountered. A final recommended IRB workflow will be created after the process is piloted and will include lessons learned, requirements for each involved institution (eg, FDA, HPHCI, participating health plans), relevant flowcharts, and recommendations for future studies.

#### NDI Application Workflow

The HPHCI will lead the NDI application development and subsequent application review by the FDA and the health plans before submission of the final application package to the NDI. The published guidelines for obtaining NDI application approval by the NDI board will inform the developed workflow [41]. The HPHCI will also work with staff at the NDI to ensure all requirements are met in accordance with the NDI guidelines. Process development may be iterative, with the NDI providing guidelines and the HPHCI subsequently working with health plans and the FDA to ensure guidelines are met. Preliminary work has identified the need for specific process development in IRB approval for the protection of human subjects, final disposition of identifiable data, and NDI-required agreements.

The HPHCI will document lessons learned from piloting the administrative workflows that will inform the development of a flexible and reusable process intended to guide future studies. The HPHCI will review the NDI and IRB stipulations encountered during this study and ensure appropriate processes and guidelines are built to accommodate them. As the NDI and IRB administrative workflows are interdependent, we will use an iterative process outlining and updating the IRB and NDI administrative workflows as new stipulations or requirements are encountered. Thus, the overall administrative workflow will include recommendations for IRB and NDI application development for use in future studies.

### Development of Distributed Process for Linkage Between Health Plan and NDI+ Data

#### Overview of the Distributed Linkage Process

**Figure 2:**
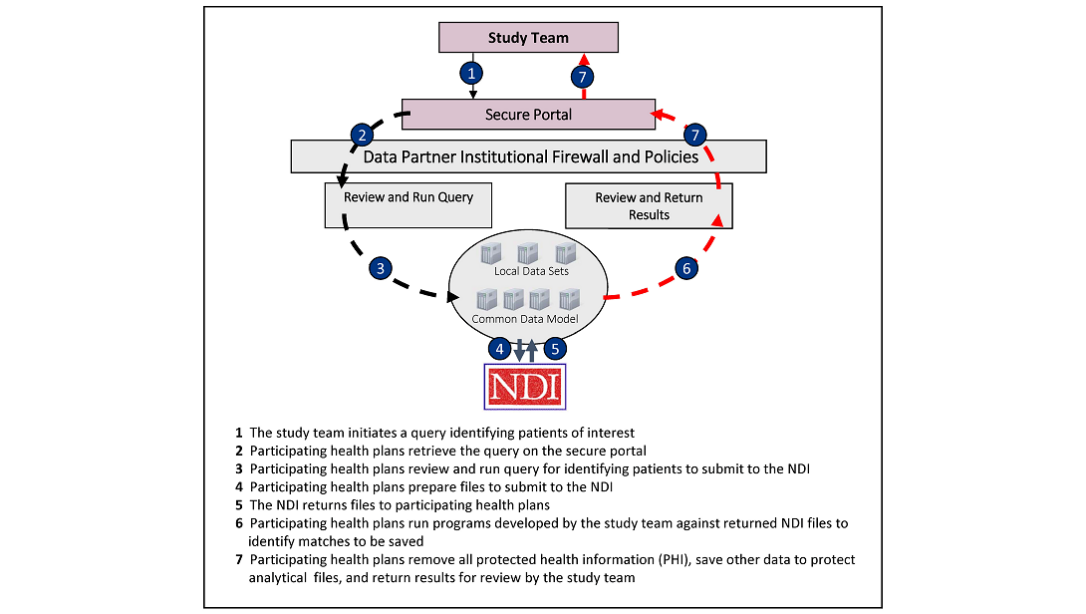
Overview of the distributed National Death Index data linkage process. NDI: National Death Index; PHI: Protected Health Information.

The HPHCI, in collaboration with the FDA and participating health plans, will develop a distributed linkage process that allows health plans to work directly with the NDI to eliminate sharing of identifiable patient information between participating health plans or with the coordinating center. The HPHCI will develop the distributed NDI+ data linkage process with input from the participating health plans and pilot the process within the study use case. Health plans will identify and submit individuals meeting specific criteria within the use case cohorts to the NDI for matching. The HPHCI will also work with each participating health plan to develop and ensure a standardized NDI+ data linkage process across databases. Figure 2 provides a high-level overview of the anticipated distributed process for linkage between health plan data and NDI+ data.

Piloting the process with the study use case will elucidate adjustments that could be made to improve efficiency and provide flexible options for future studies. We will summarize practical lessons learned from the participating health plans and the NDI. Although the NDI User’s Guide [42] describes the general process for NDI+ data linkage within a single site, the developed technical workflow will need to enable linkage to NDI+ data at multiple study sites. Accomplishing timely and standardized linkage to NDI+ data across multiple sites requires defining and implementing a set of NDI submission criteria, ensuring adequate file preparation and quality control processes across sites, standardizing the selection and retention of NDI matches, and storing information retrieved from the NDI in standardized table(s) so that study analyses can be implemented. We anticipate the following tasks will be required to build a distributed process for linkage between health plan and NDI+ data.

#### Defining NDI Submission Criteria

This project will develop, pilot, and recommend case identification and NDI submission criteria for future multicenter studies. Multimedia Appendix 1 includes the case identification and NDI submission criteria this project will use to determine which individuals will be initially selected for sending to the NDI, thereby obtaining death and cause-of-death information. We anticipate submitting patients with deaths recorded in health plan data or patients with potential deaths to the NDI for linkage. We will define potential death as health plan disenrollment between cohort entry and cohort exit plus 365 days, without subsequent reenrollment or medical utilization >60 days after disenrollment. It is possible that these NDI submission criteria will be refined or redesigned as they are piloted within the study use case. We will describe the final developed case identification and NDI submission algorithm and provide this information for use in future studies.

#### Preparing Files for Submission to the NDI

The NDI publishes information that health plans must provide to conduct an NDI+ data search as well as the required file structures in their NDI User’s Guide [42]. Health plans will need to access these required data elements from their source systems and transmit complete records to the NDI for matching. To ensure that files submitted to the NDI are of sufficient completeness, the HPHCI will develop distributed programs for local execution by the health plans to identify any potential data or formatting issues. Any lessons learned during these file preparation and quality control processes will be documented for future use and incorporation into the technical workflow.

#### Standardizing NDI+ Data Linkage Across Multiple Health Plan Databases

After files intended for submission to the NDI have been checked to ensure sufficient completeness and quality, each health plan will submit their selected health plan members for matching directly with NDI+ data. Health plan data files will be transferred to the NCHS via either password-protected encrypted CDs or a secure file transfer protocol site, according to the health plan and NCHS or NDI requirements. When NDI staff return data files directly to health plans, health plans will load the returned files to their computer servers behind their firewalls. These data sets will remain behind their firewalls and will not be shared with the HPHCI, the FDA, or other health plans. We will summarize the processes, challenges, and requirements in the technical workflow.

#### Selecting and Retaining the Best NDI Match

When the NDI performs matching, multiple possible matches for each individual submitted may be provided within the NDI-returned data files. The NDI User’s Guide [42] provides guidelines for selection and retention of NDI matches, from among multiple possible matches for each individual submitted. This requires researchers to assess the quality of each possible NDI record match listed and determine which possible matches are *best* matches. The NDI recommends a multistep process when determining the best match among possible multiple matches, including using the NDI-provided probabilistic matching scores to distinguish true matches from false matches. The HPHCI, guided by the principles within the NDI User’s Guide [42], will develop a standardized process for ascertaining and keeping confirmed or best matches locally at the participating health plan sites. This will be implemented in distributed programs to examine all possible matches and identify matches that are considered best based on specific criteria.

We will design the process to be flexible and reusable, and we anticipate a multistep process using variables within the returned NDI data files for match selection. Processes will assess the distribution of NDI-provided matching variables such as the *Status Code* (indicates NDI assessment of probability of truly being alive or dead), *Class Code* (indicates the fact that some NDI-identifying data items used in the matching criteria are more important for determining true matches than others), assessment of item-by-item matches between health plan and NDI information, and probabilistic matching scores (score for each potential match). We will implement rules for retaining NDI matches in distributed program(s).

The NDI returns a cause-of-death code only for records that rank first in the list of possible NDI matches. If our match selection process identifies a match that was not ranked first by the NDI, this record will not have the cause-of-death information in the initial NDI+ data files. In such instances, the HPHCI will work with the NDI to attain this missing cause-of-death information. However, it is possible that the NDI will be unable to supply the cause-of-death information or may have time delays for the return of this information. If this occurs, the HPHCI may not be able to include newly supplied cause-of-death information in final use case analyses and will pilot the process for requesting and attaining this information and document lessons learned.

The HPHCI will develop a proposed standardized table structure that can be used in future studies to store information retrieved from the NDI. The HPHCI will work with the health plans to develop the ultimate table structure. The data included in this table will be maintained behind each health plan’s firewall, thereby preserving the distributed nature of health plan databases. The HPHCI will document these processes and programs in a report for future use.

### Draft Use Case Specifications

#### Use Case Inclusion and Exclusion Criteria

This study will use data captured within participating health plan databases between 2000 and 2017 (or earliest or latest available health plan data) and the most recent NDI+ data available at the time of NDI application.

Cohort 1 will include new users of select antiarrhythmic medications for men aged 45 years and older and women aged 55 years and older on the date of cohort entry between 2000 and 2017 (or earliest available health plan data). The list of select antiarrhythmic medications of interest and new-user definition is described under the *Exposure Identification for the Use Case* section**.** We chose different age cutoff values for men and women because risks of all-cause mortality and SCD vary considerably by sex. The goal is to improve the specificity of mortality and specific causes of death outcomes identified through NDI+ matching. Younger individuals are less likely to experience mortality and SCD than older individuals, and within age groups, women are less likely to experience mortality and SCD than men. The risk for SCD has been shown to increase in women after the age of 55 years [43]. All-cause mortality is also rare in younger age groups. Choosing a higher age cutoff for women is intended to decrease *false-positive* matches and minimize the number of NDI submissions.

We will use the entire cohort for the all-cause mortality analysis and potentially the cardiovascular death analysis. For analyses focused on SCD, we will restrict the cohorts to individuals under the age of 75 years to maintain consistency with a study by Chung et al [27], which developed and validated a computerized algorithm to identify community originating SCD. As the risk of mortality increases with age, Chung et al [27] found death certificates to be less reliable for identifying SCD in older individuals and removed patients aged ≥75 years to minimize false positives. Although it may be difficult to capture nursing home stays within the participating health plan databases, to maintain consistency with the algorithm by Chung et al [27], we will exclude individuals with evidence of a nursing home stay in the baseline period. Cohort 1 entry will begin on an individual’s first prescription dispensing for an oral dosage form of an antiarrhythmic medication of interest that was preceded by a 365-day baseline period with medical and pharmacy benefits (gaps in enrollment <45 days bridged), during which the individual has ≥1 encounter with a diagnosis recorded in any care setting or an outpatient dispensing of any medication.

To mimic typical drug safety study situations in which no future information is available to determine medication users’ vital status, individuals with more than one episode of new use during the study period will contribute only their first episode. This study design choice also helps avoid the selection bias that use of future information may generate. The protocol allows gaps in enrollment of <45 days because it is believed that these may not represent true gaps in coverage but rather administrative changes. Index date will be the date of the first eligible dispensing for a select antiarrhythmic drug of interest.

Cohort 2 will be drawn from average-risk individuals who are not current (ie, on day of cohort entry) or past (ie, before 365 days) users of antiarrhythmic medications of interest. We will match cohort 2 at a one-to-one ratio with cohort 1 based on age, sex, and health plan. Index dates will also be matched to cohort 1. We will require individuals in cohort 2 to have a 365-day baseline period with medical and pharmacy benefits (gaps in enrollment <45 days ignored as specified above in cohort 1) and at least one medical encounter or outpatient pharmacy dispensing claim in the previous 365 days. As in cohort 1, cohort 2 will include the entire cohort for the all-cause mortality analysis and potentially the cardiovascular death analysis but will be restricted to individuals younger than 75 years and with no evidence of a nursing home stay in the baseline period for the SCD analyses. It is worth noting that individuals included in either cohort 1 or 2 may in fact have used antiarrhythmics medications outside of the study period or before enrolling in a participating health plan.

#### Use Case Exposure Definitions

We will identify select oral antiarrhythmic medications of interest using National Drug Codes. New use will be defined by excluding individuals with dispensings of class I and III antiarrhythmic drugs (all routes of administration), including amiodarone, disopyramide, dofetilide, dronedarone, flecainide, mexiletine, procainamide, propafenone, quinidine, and sotalol [44,45], in the 365-day baseline period. Individuals with dispensings of intravenous lidocaine in the 365-day baseline period will also be excluded. Baseline exposure to adenosine A1 agonists, digoxin, phenytoin, class II β-blocker agents, and calcium channel blockers (class IV) agents will be ignored.

When creating treatment episodes, we will apply a stockpiling algorithm [46] to account for the possibility that members may refill prescriptions before the end of days’ supply of their previous prescription. For example, if a member receives a 30-day dispensing for sotalol on January 1, and then receives a second 30-day dispensing on January 20, the stockpiling algorithm will adjust the second dispensing so that it starts on January 31, after the first dispensing has been used in full. The treatment episode will thus be 60 days in total, through March 1 (assuming February has 28 days). We will also implement a 14-day episode gap when creating treatment episodes to account for imperfect adherence. An episode gap is the maximum number of days of interrupted days-supply allowed between two claims for the same drugs of interest. If the number of days between when one prescription claim runs out and the next claim is smaller than or equal to the episode gap, the algorithm *bridges* these two claims to build a continuous treatment episode. However, if the number of days between the two claims of the same treatment exceeds the episode gap, the treatment episode ends at the end of the 14-day period. The episode gap is assessed after the claim service dates are adjusted by the stockpiling algorithm. Because we are interested in the risk of all-cause mortality and SCD for the class of medications in general and not individual antiarrhythmic medications, our analyses will focus on users of any antiarrhythmic medications of interest as a group, and the results will not be stratified by individual medication.

#### Use Case Follow-Up and Censoring Plan

For cohort 1, follow-up time will begin with the cohort entry-defining antiarrhythmic medication dispensing (ie, day 1 of follow-up=dispensing date) and will continue based on the treatment episode as described above. For cohort 2, follow-up time will begin on the same day as the individual’s corresponding match from the antiarrhythmic medication user cohort. Follow-up will be censored upon the earliest of the following occurrences:

1. Death or specific causes of death, as determined from NDI+ data; date of death will be the last day of follow-up (both cohorts).
2. Health plan disenrollment (gaps of enrollment <45 days will be ignored); the last day of enrollment will be the last day of follow-up (both cohorts).
3. End of database time; database end date will be the last day of follow-up (both cohorts).
4. Initiation of an antiarrhythmic medication of interest; the day before the date of medication initiation will be the last day of follow-up (cohort 2 only).
5. Excessive allowable gap between dispensings, defined as >14 days between two consecutive dispensings for a study antiarrhythmic medication of interest, the last day of follow-up included will be the end of days’ supply of the most recent dispensing of the study antiarrhythmic medication of interest +14 days (cohort 1 only).

The analysis will follow use case cohorts for death, SCD, and potentially cardiovascular death until censored. As linking to NDI+ data allows us to follow patients for survival through the end of the study period, if feasible, we will also conduct an analysis that ignores the censoring criteria and follows use case cohorts for death and SCD, and potentially cardiovascular death through the end of NDI+ data.

#### Use Case Outcomes

The primary outcomes of interest are all-cause mortality and SCD. If timeline and study resources permit, we will assess cardiovascular death as a secondary outcome of interest. Ideally, the selected outcome algorithms would: (1) facilitate the assessment of the performance or *validity* of the linkage to NDI+ data; (2) allow for comparing the incidences and incidence rates of all-cause mortality and specific causes of death with rates previously reported in the literature, or other national death information sources; and (3) use data retrieved from the NDI, and possibly information within health plan databases. To inform future studies, we will try to capture both medically attended and nonmedically attended deaths. We will identify these outcomes using NDI+ data and will evaluate each outcome separately. Although we will attempt to replicate SCD or cardiovascular death algorithms that have been previously validated by other studies, it may be necessary to modify or tailor the algorithms to data elements available within the health plan databases that have been converted into the Sentinel Common Data Model format [47]. Multimedia Appendix 2 [27,48,49] describes the operational definitions of the outcomes. We also provide the high-level details in the following paragraphs.

We will determine all-cause mortality through linkage to the NDI+ data (all deaths, including both medically attended and nonmedically attended deaths). Two algorithms for SCD will be used, both of which exclude persons aged ≥75 years. For the primary SCD definition, we will adapt an algorithm focused on community-originating events defined by Chung et al [27] for use within the health plan databases. This algorithm uses information available in claims data to exclude patients with certain conditions (Table 1 [50]) as well as cause-of-death information provided by the NDI (Table 2) [27]. The definition of secondary SCD will focus on events that occur in medical care settings. Studies examining ventricular arrhythmia diagnosis in hospital settings (ie, inpatient or emergency department) have found inpatient diagnosis codes for ventricular arrhythmia to have high positive predictive values, regardless of diagnosis code position [49,51,52]. To identify SCD outcomes originating in medical settings, we will adapt these algorithms for use within health plan databases. Secondary emergency department or inpatient diagnoses consistent with ventricular arrhythmia or sudden cardiac arrest were selected to attempt to identify events occurring in medical settings, as principal diagnosis codes would generally define conditions established after study to be chiefly responsible for admission [53]. If feasible, we may also include a sensitivity analysis exploring the principal emergency department or inpatient diagnoses consistent with ventricular arrhythmia or sudden cardiac arrest. Finally, we may examine cardiovascular death if it is determined to be feasible by the study team, and we would define cardiovascular death with cause-of-death codes typically used by national death data sources, such as the underlying cause of death consistent with a cardiovascular cause [25]. The algorithm parameters are outlined in more detail in Multimedia Appendix 2.

**Table 1:** High-risk conditions likely to be miscoded as sudden cardiac death per Ray et al^a^.

Condition	Operational definition^b^
Cancer	Diagnosis of cancer (except for nonmelanoma skin cancers) or select antineoplastic agents. Includes the following neoplasms uncertain behavior ICD-9-CM^c^ codes^d^ 235-238, *except*: 238.2 (skin), 238.9 (site unspecified), 237.70, 237.71 (neurofibromatosis), 238.4 (polycythemia vera), 238.7 (lymphoproliferative disease), and 285.22 (anemia in neoplastic disease)
HIV	Diagnosis of HIV or use of antiretroviral agents appropriate for HIV or pentamidine (also used for other major immunocompromised patients)
Renal	Diagnosis or procedure code for dialysis outside of the hospital (includes 996.73). Includes end-stage renal disease diagnosis (285.21, 585.5, 585.6), also outside of the hospital
Liver	Diagnoses 570-573
Respiratory	Diagnosis of respiratory failure, cardiorespiratory failure, or pulmonary heart disease. Also includes tracheostomy (excluding temporary), home oxygen, or home ventilator
Organ transplant	Includes kidney, heart, lung, liver, bone marrow, and pancreas. Includes complications of transplanted organ (996.8)
Serious neuromuscular	Multiple sclerosis (340), amyotrophic lateral sclerosis (335.20), Duchenne muscular dystrophy (335.21), Huntington chorea (333.4), quadriplegia, paraplegia, or spinal cord injury. Recent stroke (inpatient with primary discharge diagnosis of 430, 431, 433.x1, 434, 436) with hemiplegia/hemiparesis (342, 438.2)
Cardiovascular congenital anomalies	Common truncus (745.0) transposition great vessels (745.1), tetrology (745.2), common ventricle (745.3), endocardial cushion defect (745.6), pulmonary atresia (746.0), tricuspid atresia (746.1), hypoplastic left heart (746.7), coarctation of aorta (747.1), other anomalies of aorta (747.2), total anomalous pulmonary venous connection (747.41). A single diagnosis is sufficient for exclusion
Other congenital anomalies/childhood conditions	Sickle cell (282.6), cerebral palsy (343), spina bifida (741), Down syndrome (758.0), hydrocephalus (742.3), microcephalus (742.1), encephalocele (742.0), severe mental retardation (318.1, 318.2), cystic fibrosis
Other end-stage illness	(a) Hospice care; (b) diagnosis of coma, vegetative state, debility (799.3); (c) total parenteral nutrition, percutaneous endoscopic gastrostomy, enteral feeding, malnutrition (260, 261, 262, 263) when these are for outpatients; (d) gangrene (040, gas gangrene; 785.4 gangrene: single diagnosis sufficient); (e) intravenous medications outside of the hospital, as indicated by procedures for intravenous access outside a hospital stay period
Drug abuse	Includes all medications and drugs with abuse potential and with the exception of alcohol (unless hospitalization with primary discharge diagnosis: 291.x, 303.x, 305.0, 980.0, 980.9, E860.0, E860.1, E860.9) and tobacco. Codes are 292.0 (drug withdrawal syndrome), 304.x (drug dependence), 305.2-305.9 (drug abuse, except alcohol/tobacco, 305.9 is abuse not otherwise specified, may be nonspecific, but better to exclude), 965.01 (accidental poisoning, heroin), 969.6 (poisoning, psychodysleptic [hallucinogens]), 970.81 (cocaine poisoning, added in 2010), E8500 (heroin poisoning), E8541 (psychodysleptic poisoning)

^a^Ray et al [50].

^b^Unless otherwise indicated, codes are ICD-9-CM diagnostic codes and a 3- or 4-digit code implies inclusion of all subcodes. Further, a single diagnosis is sufficient for exclusion.

^c^ICD-9-CM: International Classification of Diseases, 9th Revision, Clinical Modification.

^d^ICD-9-CM codes will be mapped to ICD-10-CM codes during the study.

**Table 2:** Underlying cause-of-death diagnostic codes consistent with sudden cardiac death.

International Classification of Diseases, 10th Revision Code	Description
I10	essential hypertension, not otherwise specified
I11.9	hypertensive heart disease, without heart failure
I20	angina pectoris
I21	acute myocardial infarction
I22	subsequent myocardial infarction
I23	certain current complications following ST elevation and non-ST elevation myocardial infarction
I24	other acute ischemic heart disease
I25	chronic ischemic heart disease
I25.2	old myocardial infarction
I42.8, I42.9	cardiomyopathy, not otherwise specified
I46	cardiac arrest
I47.0	re-entry ventricular arrhythmia
I47.2	ventricular tachycardia
I49.0	ventricular fibrillation and flutter
I49.8	other specified cardiac arrhythmias
I49.9	cardiac arrhythmia, unspecified
I51.6	cardiovascular disease, unspecified
I51.9	heart disease, unspecified
I70.9	atherosclerosis, not otherwise specified
R96.1	death in <24 hours
R98	unattended death

#### Use Case Analytic Plan

For both cohort 1 and cohort 2, we will generate a baseline characteristics table. Table 3 includes the proposed list of baseline characteristics and Table 4 includes the initial code lists. We will examine demographic variables, health care utilization intensity measures, and select comorbid conditions during the 365-day baseline period. Expert opinion and review of the literature will inform variable selection. If feasible, we will also consider examining a claims-based measure of frailty [54].

Separately for all-cause mortality, SCD, and cardiovascular death, we will estimate the incidences and incidence rates as the number of outcome events during the observation period as defined in the outcome section below, divided by total persons in cohort (for incidences) or person-time (for incidence rates) of observation. All incidences or incidence rates will also be stratified by cohort. We will further estimate the incidences and incidence rates by age group (<65, 65-74, ≥75 [for all-cause mortality only]), sex, and cohort entry year. To facilitate comparison with previously published estimates, incidence will be presented per 1000 persons and incidence rates will be presented per 1000 person-years. For SCD, we will further estimate the incidences and incidence rates by selecting comorbidities (coronary heart disease [35,36,55,56] and diabetes mellitus [55,57,58]). If feasible, to facilitate comparisons with the literature, we will include analyses using multiple age subgroups (eg, age subgroup 1: 45-54, 55-64, 65-74, 75-84, and ≥85 years; age subgroup 2: 45-46, 47-51, 52-56, 57-61, 62-66, 67-71, 72-74; and 45-54, 55-64, 65-74) [35,64].

Although medical records, autopsy reports, ambulance, or other similar records might be used to validate death information attained from the NDI, this type of evaluation is beyond the scope of this study. If project timelines permit, we will consider two other indirect approaches to evaluate the performance of the NDI+ data linkage. The first strategy would involve comparing rates of mortality and SCD with rates previously reported in the literature. We will describe and examine the incidences and incidence rates of mortality and SCD in the use case cohorts and compare them with estimates previously reported in the literature. This comparison will provide indirect evidence for outcome definition accuracy. For all-cause mortality, we will compare our estimated incidence rates with those from the CDC Wonder data [65]. For SCD, we will compare the incidence rates estimated in cohort 1 with the range of incidence rates reported in the literature (Table 5). In general, we will examine and compare the incidences and incidence rates in cohort 2 with national data sources such as CDC Wonder and studies included in the literature because such data sources and studies focus on the overall population and are thus are comparable with our cohort 2.

**Table 3:** Baseline characteristics associated with users of antiarrhythmic medications (cohort 1) and among the average-risk population (cohort 2) identified at participating health plans, 2000 to 2017 or latest health plan and National Death Index Plus data availability.

Demographics			Cohort 1^a^	Cohort 2^a^
**Age groups (<65, 65-74, ≥75)**				
	Mean age, in years (±SD)		N/A^c^	N/A
	Median age, in years (±SD)		N/A	N/A
	Sex, % female		N/A	N/A
**Health care utilization intensity measures during the baseline period**				
	#hospitalizations		N/A	N/A
	#emergency department visits		N/A	N/A
	#ambulatory care visits		N/A	N/A
	#unique medications dispensed		N/A	N/A
**Comorbid conditions, identified during the baseline period**				
	Arrhythmia/conduction disorder, by type		N/A	N/A
		Atrial fibrillation and flutter	N/A	N/A
		Paroxysmal ventricular tachycardia	N/A	N/A
		Ventricular fibrillation and flutter	N/A	N/A
		Paroxysmal supraventricular tachycardia	N/A	N/A
		Unspecified paroxysmal tachycardia	N/A	N/A
		Premature beats	N/A	N/A
		Other specified or unspecified cardiac dysrhythmia	N/A	N/A
	Cerebrovascular disease		N/A	N/A
	Coronary heart disease		N/A	N/A
	Diabetes mellitus		N/A	N/A
	Heart failure/cardiomyopathy		N/A	N/A
	Cardioverter-defibrillator/pacemaker		N/A	N/A
	Hyperlipidemia		N/A	N/A
	Hypertension		N/A	N/A
	Kidney disease		N/A	N/A
	Circulatory system disease		N/A	N/A
	Seizure disorder		N/A	N/A
	Smoking^b^		N/A	N/A
	Obesity^b^		N/A	N/A
**Charlson comorbidity score**				
	0		N/A	N/A
	1		N/A	N/A
	≥2		N/A	N/A
**Risk of Torsades de pointes (TdP), per CredibleMeds [28]**				
	Known risk		N/A	N/A
	Possible risk		N/A	N/A
	Conditional risk		N/A	N/A
	To be avoided by congenital long QT patients		N/A	N/A

^a^This table represents planned study analyses, and cells are blank because analyses are not yet complete.

^b^Although these covariates are often not well-captured in claims data, given the importance of these factors we will include them with the understanding under capture of these elements is expected within source data.

^c^N/A: Not yet available

**Table 4:** International Classification of Diseases, 9th Revision, Clinical Modification, diagnosis, and procedure codes for identifying comorbidities and other conditions.^a^

Baseline table conditions		Codes
Atrial fibrillation and flutter		ICD-9^b^-CM: 427.31 and 427.32
Paroxysmal ventricular tachycardia		ICD-9-CM: 427.1
Ventricular fibrillation and flutter		ICD-9-CM: 427.4X
Paroxysmal supraventricular tachycardia		ICD-9-CM: 427.0
Unspecified paroxysmal tachycardia		ICD-9-CM: 427.2
Premature beats		ICD-9-CM: 427.6X
Other specified or unspecified cardiac dysrhythmia		ICD-9-CM: 427.8X or 427.9X
Cerebrovascular disease		ICD-9-CM: 430.X-432.X 433.01, 433.11, 433.21, 433.31, 433.81, 433.91, 434.x, 436 362.34, 433.00, 433.10, 433.20, 433.30, 433.80, 433.90, 435.x, 437.0, 437.1, 437.9, 438.x 38.11, 38.12, 38.41, 38.42 325.X, 437.6 781.4, 784.3, 997.0
Coronary heart disease [35,36,55,56]		ICD-9-CM: 410.XX, 412.XX, 412, 413.X, 414.XX
Diabetes mellitus [55,57,58]		ICD-9-CM: 250.XX
Heart failure/cardiomyopathy [35,59,60]		ICD-9-CM: 402.X1, 404.X1, 404.X3, 428.XX
Cardioverter-defibrillator/pacemaker		ICD-9-CM: 996.01, 996.04, V45.X, V53.31, V53.32; ICD-9-CM Volume 3 procedure codes: 00.50─00.54, 37.7X, 37.8X, 37.94, 37.95, 37.96, 37.97, 37.98, 89.45─89.49 CPT-4^c^ Category II codes: 00530, 33200─33249, 33262─33264, 93280, 93288, 93294, 93296, 93297, 93640, 93641, 93642 CPT-4 Category III codes: 0319T─0328T Healthcare Common Procedure Coding System codes (HCPCS): C1721, C1722, C1777, C1779, C1785, C1786, C1882, C1895, C1896, C1898, C1899, C2619, C2620, C2621, E0610, E0615, E0617, G0297, G0298, G0299, G0300, G0448, K0606, K0607, K0608, K0609
Hyperlipidemia		ICD-9-CM: 272.0X, 272.1X, 272.2X, 272.3X, 272.4X, 272.7X
Hypertension		ICD-9-CM: 401–405 (excluding 402.01, 402.11, 402.91)
Chronic kidney disease [58,61,62]		ICD-9-CM: 585.3, 585.4, 585.5
Circulatory system disease, thereby capturing rheumatic fever, rheumatic heart disease, hypertensive disease, ischemic heart disease, diseases of pulmonary circulation, other heart disease, cerebrovascular disease, arterial disease, and venous disease		ICD-9-CM: 390.X–459.X
Seizure disorder		ICD-9-CM: 345x, 780.3x (not 780.31)
Smoking tobacco [55]^e^		Presence of any the following codes on any claim type: ICD-9-CM: 305.1, 649.0X, 989.84, V15.82; CPT-I: 83887, 99406, 99407; CPT-II: 1034F, 1035F, 4000F, 4001F, 4004F; HCPCS: C9801, C9802, G0375, G0376, G0436, G0437, G8093, G8094, G8402, G8403, G8453, G8454, G8455, G8456, G8688, G9016, S4990, S4991, S4995, S9075, S9453; NDC^d^: nicotine replacement, varenicline, Zyban (brand only)
Obesity [55,63]^e^		278.0X
**Conditions included in the SCD^f^ subgroup analyses**		
	Coronary heart disease [35,36,55,56]	410.XX, 412.XX, 412, 413.X, 414.XX
	Diabetes mellitus [55,57,58]	250.XX

^a^Codes will be mapped to ICD-10-CM (ICD-10: International Classification of Diseases, 10th Revision) codes during the study

^b^ICD-9-CM: International Classification of Diseases, 9th Revision.

^c^CPT-4: Current Procedural Terminology-4.

^d^NDC: National Drug Code.

^e^Although obesity and smoking are often not well-captured in claims data, we will include them with the understanding under capture of these elements is expected within source data.

^f^SDC: sudden cardiac death.

**Table 5:** Published incidences or incidence rates of sudden cardiac death and all-cause mortality among users of antiarrhythmic medications and among the average-risk population.

Patient characteristics			Events per person or person-years, and/or risk of sudden cardiac death by patient characteristics		Events per person or person-years or risk of all-cause mortality by patient characteristics^a^	
			Antiarrhythmic medication users^b^	Average-risk population, without respect to antiarrhythmic use	Antiarrhythmic medication users	Average-risk population, without respect to antiarrhythmic use
Overall			N/A	0.5-1.5/1000 persons, Deo et al [66], Chugh et al [36], Straus et al [67]	N/A^c^	N/A
Female			N/A	Female<male, Zheng et al [43], Kannel et al [68], Stecker et al [37]; Beginning at age 35, incidence increases monotonically until age 85 (Zheng et al [43], Chugh et al [36], Straus et al [67])	N/A	N/A
	55-64 years		N/A	1.0/1000 persons	N/A	N/A
	65-74 years		N/A	2.8/1000 persons	N/A	N/A
Male			N/A	Male>female, Zheng et al [43], Kannel et al [59], Stecker et al [37]; Beginning at age 35, incidence increases monotonically until age 85 (Zheng et al [43], Chugh et al [36], Straus et al [67])	N/A	N/A
	45-54 years		N/A	1.2/1000 persons	N/A	N/A
	55-64 years		N/A	2.8/1000 persons	N/A	N/A
	65-74 years		N/A	6.0/1000 persons	N/A	N/A
Year			N/A	Given that sudden cardiac death incidence declined from 1979-1998 [69], it may be reasonable to expect a small decline in incidence from 2001-2002 to 2009-2010. This is likely driven by a reduction in coronary heart disease. Yet, any small decline could be halted by the increasing incidence of heart failure [70]	N/A	N/A
	1990-1995		N/A	1.0/1000 person-years (for 1990s) [71]	N/A	N/A
	1996-1999		N/A	0.91-1.0/1000 persons [67]	N/A	N/A
	2000-2004		N/A	0.79/1000 persons [67]	N/A	N/A
	2005-2009		N/A	N/A	N/A	N/A
	2010-2014		N/A	N/A	N/A	N/A
	2015-2017		N/A	N/A	N/A	N/A
**Comorbidities**					N/A	
	**Coronary heart disease**			2-12X increased risk, Chugh et al [36], Kannel et al [56,59], Albert et al [72]	N/A	N/A
		Presence	N/A	4.6-25.1/1000 persons	N/A	N/A
		Absence	N/A	1.5-3.6/1000 persons	N/A	N/A
	**Diabetes mellitus**			2-3 times increased risk, Jouven et al [73,74], Albert et al [72], Vasiliadis et al [58]; 1.3/1000 person-years in sulfonylurea users Leonard et al [75]	N/A	N/A
		Presence	N/A	N/A	N/A	N/A
		Absence	N/A	N/A	N/A	N/A

^a^Estimates from CDC Wonder or other national death data sources.

^b^Estimates located at the time or protocol development were included, blank cells indicate no available information at the time of protocol development.

^c^N/A: Not yet available.

The second strategy would be to examine the concordance between NDI data and health plan death data. Several participating health plans collect death information through linkage with the state death records. If timeline and resources permit, this project will attempt to identify time periods in which death information is considered well populated within each health plan and examine the concordance of this information with information attained through linkage to NDI data. At health plans that do not attain death information from state death records, if timeline and resources permit, we will consider examining discharge disposition (ie, discharged expired) for in-hospital deaths included in health plan databases, and comparing this information with NDI data. Although we expect agreement between both data sources, such comparisons will assist in any evaluations of matching with NDI data and would also provide indirect evidence for accuracy (Table 6).

**Table 6:** Example concordance matrix, all-cause mortality (to be repeated for each health plan and time period of interest^a^).

NDI^b^ data	Health plan data	
	Health plan 1 death=yes^c^	Health plan 1 death=no^c^
NDI death=yes	A	C
NDI death=no	B	D

^a^Death data within the health plan databases are known to be incomplete. Time period of interest will be time periods in which participating health plans are confident in the completeness of their death data. Additional stratifications, such as stratifying results by data source (eg, hospital discharge disposition) may be conducted.

^b^NDI: National Death Index.

^c^No gold standard, can only describe concordance and discordance (ie, “a” and “d” concordance, “b” and “c” discordant).

#### Proposed Use Case Workflow

Below, we summarize a high-level overview of steps to execute the use case.

1. Study team will finalize the following:
  Use case specifications
  Criteria for NDI patient record submission
  The limited set of identifiable data elements needed for NDI+ matching
  Analytic plan
2. The HPHCI will develop a cohort identification program that will query health plan databases formatted in the Sentinel Common Data Model. This program will identify individuals who meet the criteria entry into the cohorts as well as for matching with the NDI at the participating health plans; the program will be distributed to participating health plans for local execution.
3. Participating health plans will populate files to be sent directly to the NDI from their operational data source with the NDI required patient identifiers (eg, name, date of birth, age, social security number).
4. The HPHCI will develop a data quality assurance and check program that will ensure that the data files to be sent to the NDI are completely populated, meet NDI’s minimal criteria as eligible for matching, and are correctly formatted. The program will be distributed to participating health plans for local execution.
5. Participating health plans will individually submit the necessary quality-checked data files to the NDI.
6. The NDI will conduct matching activities and return files to health plans.
7. The HPHCI will develop a program to remove all identifiable data, identify matches to be saved, and create analytic files with minimally necessary information from health plan data and the NDI. The program will be distributed to participating health plans for local execution.
8. The HPHCI will develop an analytic program to generate information necessary to conduct the statistical analysis for the use case. The program will be distributed to participating health plans for local execution, and only summary-level information will be shared between health plans and the coordinating center.
9. The HPHCI will retrieve output produced by health plans and complete the statistical analysis.
10. The HPHCI will lead the writing of the final project report and standard operating procedures.

## Results

We will use the linked health plan and NDI+ data sets to estimate the incidence and incidence rate of mortality and specific causes of death within the use case and compare the results with previously reported estimates. These comparisons provide an opportunity to assess the performance of the developed NDI+ linkage approach and lessons to future studies requiring NDI+ linkage in distributed database settings. This study is approved by the Harvard Pilgrim Health Care IRB in Boston, MA. We will present results and the reusable NDI+ linkage approach to the FDA, at academic conferences, and publish in peer-reviewed journals. We have attained NDI approval and are summarizing the administrative processes that we developed and implemented for use in other studies. Currently, the study team is in the process of developing and testing the distributed NDI+ linkage process as described above and anticipates having initial results in early 2021.

## Discussion

### Use Case Limitations

Given that the outcomes of death, SCD, and cardiovascular death could be rare in the general population; large cohorts will be required to adequately address the use case. Although we anticipate potentially large available sample sizes within the use case, estimates of incidences and incidence rates in small subgroups may be imprecise. If it is not feasible to perform linkage for all the identified individuals, we will develop a sampling scheme that will still allow us to pilot the linkage methods.

The incidences and incidence rates estimated from our study may not be directly comparable with those reported in the literature. For example, our proposed use case exclusion conditions and matching of persons in cohort 2 with persons of cohort 1 by age, sex, health plan, and index dates (thereby making the population in cohort 2 more similar to the antiarrhythmic medication users in cohort 1), may make our population of interest different from other populations studied previously. In addition, privately insured patients may have lower mortality rates compared with the general population owing to better health care access. Due to these anticipated differences, the comparison between the incidences and incidence rates derived from our study and the literature-reported estimates will be performed qualitatively.

Some of the outcome algorithms used in this study have been validated in other data sources but have not been validated specifically within the participating health plan databases. For example, the SCD algorithm by Chung et al [27] was originally developed and implemented within a population including Tennessee Medicaid recipients aged 30-74 years. While the participating health plans in this study include mainly commercially insured populations, Medicaid beneficiaries included in the study by Chung et al may be different (eg, more vulnerable, economically disadvantaged). However, in our study, one participating health plan also provides Tennessee Medicaid data, and thus analyses stratified by health plan may inform potential population differences. In addition, the Chung et al study relied on both death certificate data and state hospital discharge data when developing a computerized algorithm to identify SCD. Although not all information included in the Chung et al study is available to participating health plans, the selected algorithms can be adapted to utilize data elements available within health plan data. The potential inability to replicate validated computerized algorithms developed in other data sources in their entirety is a study limitation.

Health plan disenrollment will be used as a proxy to select individuals for linkage to NDI+ data. Most individuals who disenroll from their health plans have not died but instead have lost or changed their insurance coverage. If individuals in an average-risk cohort are healthier and more likely to change health insurance plans, they may have higher rates of disenrollment than antiarrhythmic medication users. These higher rates of disenrollment are unlikely to reflect death and may lead to a disproportionate number of submissions to the NDI that do not result in a death record. We expect that the incidence of death and SCD will be low and disenrollment rates will be high (approximately 20%-30% per year). Therefore, we expect that our NDI+ data linkage activity will yield false positives. However, given the goal of this project is to determine an algorithm for identifying individuals to submit to NDI in future studies, lessons learned concerning false positives during analyses examining concordance between health plan death data and NDI data as well as ways to refine the disenrollment algorithm will inform future NDI+ data linkage studies.

In general, study results will be highly dependent on the quality of the NDI+ data linkage. Some identifiers that would be highly desirable to use as keys for linkage may not be uniformly available across all health plans. For example, provision of social security number information to the NDI will likely increase the number of correct matches. However, social security number information is not always complete in health plans. A lack of social security number submittal could result in a greater number of multiple matches returned by the NDI, which requires resolution and selection. The study team is designing strategies to optimize the selection of the best match. However, regardless of whether a social security number is submitted, it is possible that an incorrect match could be selected. In addition, if personal identifiers submitted by the health plans are incorrect, mismatches between health plan and NDI+ data could also occur. Such mismatches will most likely result in misclassifying patients who are dead as alive (ie, unable to locate a death in NDI+ data). The study team has anticipated these potential issues and is designing quality assurance steps where possible. To inform future studies, we will summarize lessons learned about ways to maximize the quality of the NDI+ data linkage.

### Study Strengths

The NDI is currently the best data source of death and cause-of-death information for large-scale population-based epidemiologic studies in the United States. We anticipate the development of standardized processes to attain and analyze death and cause-of-death information from the NDI will provide avenues for multisite research networks to efficiently obtain more complete death information. As many health plans that participate in multisite research networks do not have complete capture of out-of-hospital deaths or cause-of-death information, the ability to efficiently attain this information from the NDI may provide opportunities to answer a wider variety of mortality-related research questions. We also anticipate that our newly developed NDI+ linkage methods will enhance the FDA’s ability to answer mortality-related safety questions in distributed networks.

Although conducted independently of the Sentinel Initiative, our study will leverage the infrastructure of a well-known distributed network, the FDA Sentinel System [7,8], to develop and test reusable administrative and technical processes for linking multiple health plan databases with NDI+ data. Leveraging the Sentinel System infrastructure will ensure that health plan databases are standardized and research ready. As our study sites are health plans that participate in the Sentinel System, administrative processes or NDI+ data linkage programs we will develop could be reused by the Sentinel System as well as other multisite studies using distributed research networks. As the Sentinel System publishes its common data model publicly [7,8] and in some instances provides translation code to help certain data sources with data conversion, other researchers would have the ability to directly transform other health plan databases into the Sentinel Common Data Model and directly use any developed NDI+ data linkage programs from this study for NDI+ data linkage. In addition, we will test our newly developed NDI+ data linkage methods among a diverse group of participating health plans (ie, national insurers, regional health plans, and integrated delivery systems, which cover both commercial and public insurance programs). We anticipate that our testing will ensure that developed NDI+ data linkage processes will be applicable to multiple settings.

Another strength of this study is our focus on developing a distributed process for NDI+ data linkage in multisite research studies. A distributed approach allows individual study sites to maintain physical and operational control over their electronic health data behind their respective firewalls, thus promoting data sharing by protecting patient privacy, data security, and proprietary interests [9-11]. We will develop methods that will allow health plans to work directly with the NDI and eliminate sharing of identifiable patient information between participating health plans or the coordinating center.

Finally, we chose our antiarrhythmic medications use case to robustly test the NDI+ data linkage processes within a cohort at high risk of death (antiarrhythmic medication users) and a cohort at average risk of death (nonusers matched by age and sex to antiarrhythmic medication users). This use case should provide sufficient sample sizes for patients who are dead and alive. To indirectly validate our newly developed linkage methods, we plan to examine the concordance between NDI data and health plan death data as well as compare rates of mortality and SCD with rates previously reported in the literature. Information we will gather as part of these indirect validation activities will provide some metrics for the performance of our NDI+ data linkage methods.

### Anticipated Study Contributions

We anticipate this project to provide future studies with a tested administrative workflow that facilitates efficient, coordinated, multicenter IRB review and approval for linking health plan data with NDI+ data in accordance with the revised Common Rule. We will also provide recommendations for completing a successful NDI application, along with lessons learned that may help future studies navigate the process more efficiently. We will develop a standardized and reusable distributed technical process for efficiently attaining and analyzing death and cause-of-death information from the NDI across multiple health plan databases without sharing protected health information between health plans or with the coordinating center. Our study will also provide considerations for determining which patients to submit to the NDI for matching. We will leverage lessons learned by developing and testing our NDI+ data linkage methods with the goal of improving the ability to answer mortality-related research questions within multisite studies based in distributed data networks.

## Appendix

Multimedia Appendix 1 Proposed National Death Index submission criteria to be used to determine which individuals will be initially selected for sending to the NDI, thereby obtaining death and cause-of-death information.

Multimedia Appendix 2 Operational definitions of outcomes of interest in the use case.

## Abbreviations

FAERS: FDA Adverse Event Reporting System
FDA: United States Food and Drug Administration
FWA: Federalwide Assurance
HPHCI: Harvard Pilgrim Health Care Institute
ICD-9: International Classification of Diseases, 9th Revision
ICD-10: International Classification of Diseases, 10th Revision
IRB: institutional review board
NCHS: National Center for Health Statistics
NDI: National Death Index
NDI+: National Death Index Plus
SCD: sudden cardiac death
SSA: Social Security Administration
