# Supplementary material for: Developing a Standardized and Reusable Method to Link Distributed Health Plan Databases to the National Death Index: Methods Development Study Protocol
Source: JMIR Res Protoc. 2020 Nov 2;9(11):e21811. doi: 10.2196/21811 (PMC7669437; doi:10.2196/21811)
Supplement: Multimedia Appendix 1 [file resprot_v9i11e21811_app1.pptx]

## Slide 1
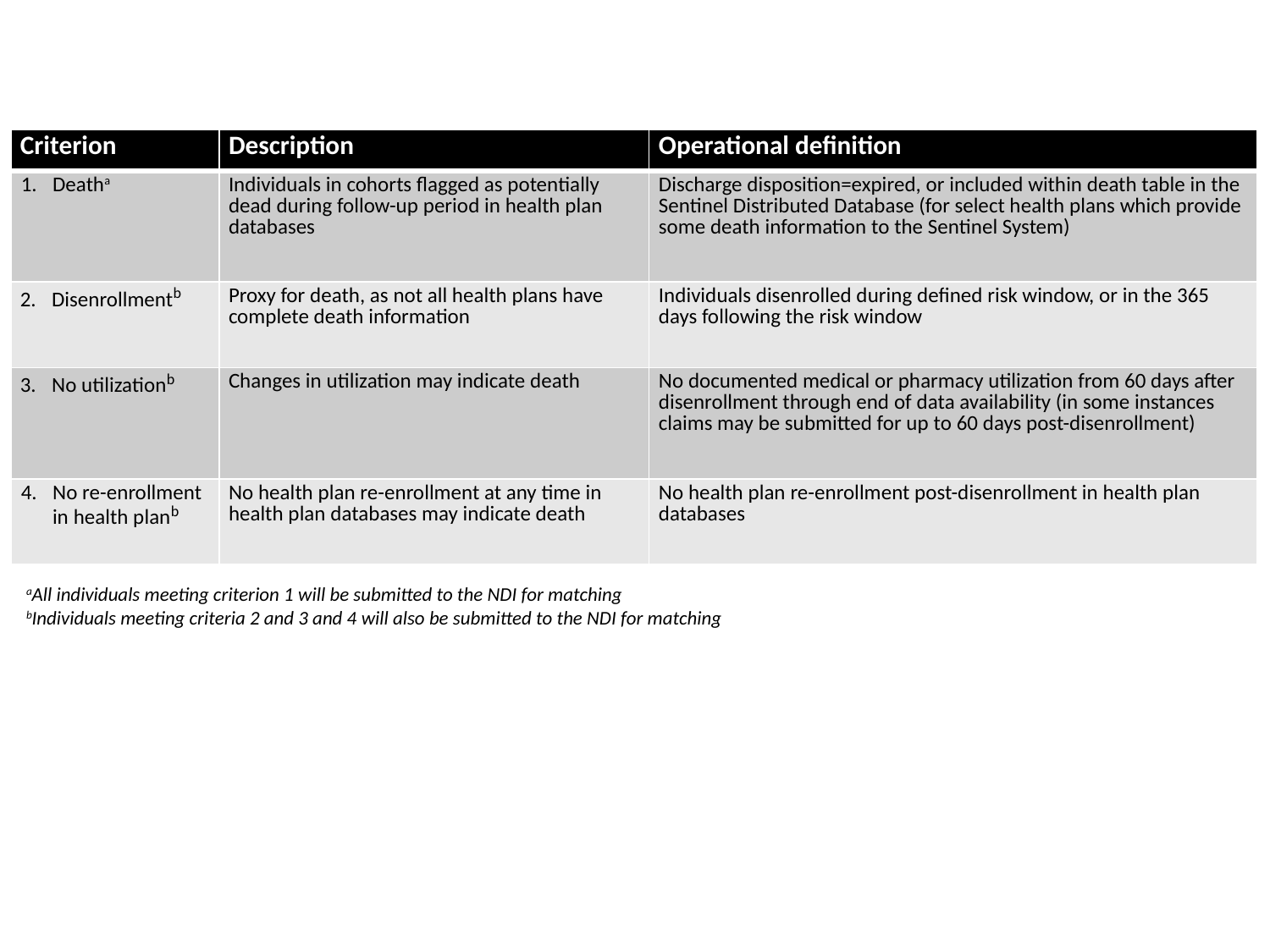

| Criterion | Description | Operational definition |
| --- | --- | --- |
| Deatha | Individuals in cohorts flagged as potentially dead during follow-up period in health plan databases | Discharge disposition=expired, or included within death table in the Sentinel Distributed Database (for select health plans which provide some death information to the Sentinel System) |
| Disenrollmentb | Proxy for death, as not all health plans have complete death information | Individuals disenrolled during defined risk window, or in the 365 days following the risk window |
| No utilizationb | Changes in utilization may indicate death | No documented medical or pharmacy utilization from 60 days after disenrollment through end of data availability (in some instances claims may be submitted for up to 60 days post-disenrollment) |
| No re-enrollment in health planb | No health plan re-enrollment at any time in health plan databases may indicate death | No health plan re-enrollment post-disenrollment in health plan databases |
aAll individuals meeting criterion 1 will be submitted to the NDI for matching
bIndividuals meeting criteria 2 and 3 and 4 will also be submitted to the NDI for matching
