# Supplementary material for: Developing a Standardized and Reusable Method to Link Distributed Health Plan Databases to the National Death Index: Methods Development Study Protocol
Source: JMIR Res Protoc. 2020 Nov 2;9(11):e21811. doi: 10.2196/21811 (PMC7669437; doi:10.2196/21811)
Supplement: Multimedia Appendix 2 [file resprot_v9i11e21811_app2.pptx]

## Slide 1
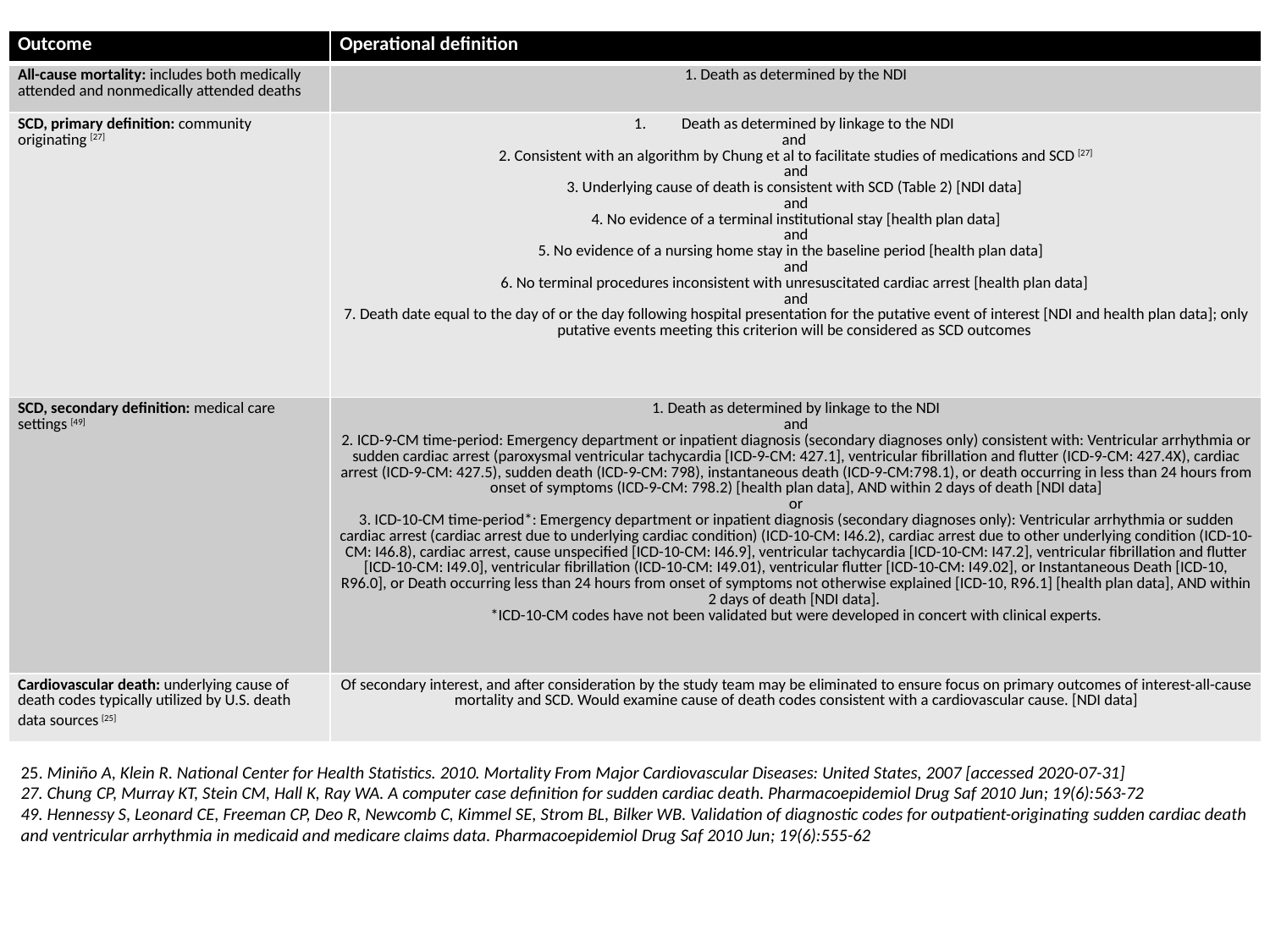

| Outcome | Operational definition |
| --- | --- |
| All-cause mortality: includes both medically attended and nonmedically attended deaths | 1. Death as determined by the NDI |
| SCD, primary definition: community originating [27] | Death as determined by linkage to the NDI and 2. Consistent with an algorithm by Chung et al to facilitate studies of medications and SCD [27] and 3. Underlying cause of death is consistent with SCD (Table 2) [NDI data] and 4. No evidence of a terminal institutional stay [health plan data] and 5. No evidence of a nursing home stay in the baseline period [health plan data]    and 6. No terminal procedures inconsistent with unresuscitated cardiac arrest [health plan data] and 7. Death date equal to the day of or the day following hospital presentation for the putative event of interest [NDI and health plan data]; only putative events meeting this criterion will be considered as SCD outcomes |
| SCD, secondary definition: medical care settings [49] | 1. Death as determined by linkage to the NDI and 2. ICD-9-CM time-period: Emergency department or inpatient diagnosis (secondary diagnoses only) consistent with: Ventricular arrhythmia or sudden cardiac arrest (paroxysmal ventricular tachycardia [ICD-9-CM: 427.1], ventricular fibrillation and flutter (ICD-9-CM: 427.4X), cardiac arrest (ICD-9-CM: 427.5), sudden death (ICD-9-CM: 798), instantaneous death (ICD-9-CM:798.1), or death occurring in less than 24 hours from onset of symptoms (ICD-9-CM: 798.2) [health plan data], AND within 2 days of death [NDI data] or 3. ICD-10-CM time-period\*: Emergency department or inpatient diagnosis (secondary diagnoses only): Ventricular arrhythmia or sudden cardiac arrest (cardiac arrest due to underlying cardiac condition) (ICD-10-CM: I46.2), cardiac arrest due to other underlying condition (ICD-10-CM: I46.8), cardiac arrest, cause unspecified [ICD-10-CM: I46.9], ventricular tachycardia [ICD-10-CM: I47.2], ventricular fibrillation and flutter [ICD-10-CM: I49.0], ventricular fibrillation (ICD-10-CM: I49.01), ventricular flutter [ICD-10-CM: I49.02], or Instantaneous Death [ICD-10, R96.0], or Death occurring less than 24 hours from onset of symptoms not otherwise explained [ICD-10, R96.1] [health plan data], AND within 2 days of death [NDI data]. \*ICD-10-CM codes have not been validated but were developed in concert with clinical experts. |
| Cardiovascular death: underlying cause of death codes typically utilized by U.S. death data sources {25] | Of secondary interest, and after consideration by the study team may be eliminated to ensure focus on primary outcomes of interest-all-cause mortality and SCD. Would examine cause of death codes consistent with a cardiovascular cause. [NDI data] |
25. Miniño A, Klein R. National Center for Health Statistics. 2010. Mortality From Major Cardiovascular Diseases: United States, 2007 [accessed 2020-07-31]
27. Chung CP, Murray KT, Stein CM, Hall K, Ray WA. A computer case definition for sudden cardiac death. Pharmacoepidemiol Drug Saf 2010 Jun; 19(6):563-72
49. Hennessy S, Leonard CE, Freeman CP, Deo R, Newcomb C, Kimmel SE, Strom BL, Bilker WB. Validation of diagnostic codes for outpatient-originating sudden cardiac death and ventricular arrhythmia in medicaid and medicare claims data. Pharmacoepidemiol Drug Saf 2010 Jun; 19(6):555-62
